# Adolescents with and without idiopathic scoliosis have similar self-reported level of physical activity: a cross-sectional study

**DOI:** 10.1186/s13013-016-0082-y

**Published:** 2016-07-28

**Authors:** Elias Diarbakerli, Anna Grauers, Hans Möller, Allan Abbott, Paul Gerdhem

**Affiliations:** 1 Department of Clinical Sciences, Intervention and Technology (CLINTEC), Karolinska Institutet, Karolinska University Hospital, K54, SE-141 86 Stockholm, Sweden; 2 Department of Orthopaedics, Karolinska University Hospital, Stockholm, Sweden; 3 Department of Orthopaedics, Sundsvall and Härnösand County Hospital, Sundsvall, Sweden; 4 Faculty of Health Science and Medicine, Bond University, Gold Coast, Australia; 5 Department of Medical and Health Sciences, Division of Physiotherapy, Faculty of Health Sciences, Linköping University, Linköping, Sweden

**Keywords:** Adolescent, Scoliosis, Physical activity, International Physical Activity Questionnaire, Metabolic Equivalent Task

## Abstract

**Background:**

Little is known about physical activity levels in individuals with idiopathic scoliosis. The aim of this study was to describe the level of physical activity in adolescents with and without idiopathic scoliosis.

**Methods:**

Two hundred thirty-nine adolescents, median (25th, 75th percentile) age 16.0 (14.4, 17.6) with idiopathic scoliosis and 58 randomly recruited population-based individuals without scoliosis aged 14.6 (12.8, 16.3) participated. The 239 idiopathic scoliosis patients consisted of 88 untreated, 43 previously braced, 36 with ongoing brace-treatment and 72 surgically treated individuals. Main outcome measure was the proportion achieving at least moderate activity level, as estimated by the International Physical Activity Questionnaire short form (IPAQ-SF). Other outcome measures were Metabolic Equivalent Task (MET) minutes/week, time spent sitting, spare time activity level and sporting activities. Statistical analyses were adjusted for age and sex.

**Results:**

The proportion of individuals with scoliosis with moderate activity level was 180 out of 239 (75 %) and for individuals without scoliosis 49 out of 58 (85 %) (*p* = 0.14). Median MET-minutes/week (25th,75th percentile) was for individuals with scoliosis scoliosis 1977 (840,3777) and for individuals without scoliosis 2120 (887,4598) (*p* = 0.11). Sporting activities did not differ (*p* = 0.28). The ongoing brace-treatment group had a significantly higher proportion of individuals categorizing themselves at high spare time activity level compared to the surgically treated and previously braced individuals (*p* = 0.046). No difference was seen between the treatment groups regarding the proportion achieving moderate activity (*p* = 0.11) and sporting activities (*p* = 0.20). Median MET minutes/week was 2160 (794,3797) for the untreated group, 989 (661,2706) for the previously braced group, 2055 (1010, 4026) for the surgery group and 2106 (990,4480) for the ongoing brace-treatment group (*p* = 0.031).

**Conclusion:**

Adolescents with idiopathic scoliosis show similar levels of self-reported physical activity as individuals without idiopathic scoliosis. Bracing and surgery do not appear to inhibit physical activity.

## Background

The prevalence of idiopathic scoliosis has been reported to be 3 % in children [1]. Out of these, about one tenth require treatment with brace or surgery [1]. Both bracing and surgery restrict motion, which may have an impact on muscle function and physical activity [2, 3]. A previous cross-sectional study in adults with scoliosis found that they perform less sporting activities due to back pain and functional disturbance compared to age-matched controls [4].

There is a possibility that surgical or brace treatment could restrict activity, and especially physical activity in daily life. Furthermore, lower bone density has been reported among adolescents with scoliosis when compared to controls [5]. Bone density itself correlates with physical activity [6], and therefore, physical activity is interesting to map in adolescents with scoliosis.

General sporting activities are recommended to be performed in patients with idiopathic scoliosis in order to benefit mental and physical well-being. However, sporting activities themselves should not be prescribed as treatment for idiopathic scoliosis [7].

The aim of this study was to compare self-reported physical activity level among adolescents with and without idiopathic scoliosis. Furthermore, a subgroup analysis was done comparing untreated, brace treated, previously braced and surgically treated individuals.

## Methods

### Individuals with idiopathic scoliosis

**Fig. 1:**
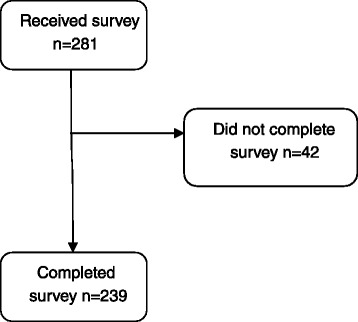
Flow chart of the individuals with idiopathic scoliosis in the study

Patients referred to and under observation or treatment for idiopathic scoliosis at any of six Swedish Orthopaedic Departments (Karolinska University Hospital in Stockholm, Skåne University Hospital, Sahlgrenska University Hospital, Umeå University Hospital, Sundsvall and Härnösand County hospital and Linköping University Hospital) were considered eligible to participate in this cross-sectional study. Inclusion criteria were the presence of an idiopathic scoliosis with a Cobb angle of ten degrees or more, with no signs or symptoms associated with a scoliosis of a non-idiopathic origin. 281 individuals were invited between September 23^rd^, 2010 and May 20^th^, 2015; 239 complied by answering the survey; 88 were untreated, 36 had an ongoing brace-treatment, 43 had terminated bracing, and 72 had been treated with surgery. A total of 19 out of 36 (53 %) individuals in the ongoing brace-treatment group wore a fulltime Thoraco-Lumbo-Sacral-Orthosis (TLSO), while the rest wore a hyperextension nighttime brace. Figure 1 shows the inclusion process.

### Individuals without idiopathic scoliosis

**Fig. 2:**
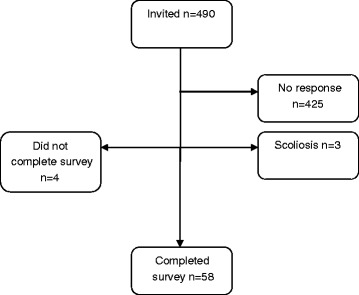
Flow chart of the individuals without idiopathic scoliosis in the study

Individuals aged ten to 15 years were randomly recruited from the Stockholm County population register, which comprises the population of Stockholm County, and individuals aged 16 to 18 years were randomly recruited from the Swedish population register. Those aged 16 to 18 years, or their legal guardians (relevant for those aged ten to 15 years) were contacted by mail between June 11^th^, 2012 and June 8^th^, 2015, and were invited to answer the same questionnaire as the individuals with idiopathic scoliosis. The individuals without idiopathic scoliosis were contacted up to three times if they did not respond to the first mailing; they did not undergo physical examination. Individuals were excluded if scoliosis was indicated in the survey. In total, 58 out of 490 invited individuals without idiopathic scoliosis participated. The non-participants consisted of four that did not complete the survey, 425 that did not respond, and three that had a radiographically diagnosed scoliosis and were therefore excluded. Figure 2 shows the inclusion process.

### Questionnaire data

The self-reported International Physical Activity Questionnaire short form (IPAQ-SF) instrument was used to determine physical activity levels [8]. Vigorous activity, moderate activity, and walking, performed in the preceding seven days for at least ten minutes were registered. Activities exceeding 180 min per day were re-coded to 180 min [9]. IPAQ-SF does not assess specific types of activity beyond intensity level.

### Achieving weekly moderate activity

A categorization of individuals was made based on whether or not they achieved at least moderate activity during the last seven days, which was considered as the primary outcome variable. To be categorized as an achiever of moderate activity, individuals needed to fulfill one of the following three criteria: three or more days of vigorous activity of at least 20 min per day, five or more days of moderate-intensity activity and/or walking of at least 30 min per day, or five or more days of any combination of walking, moderate-intensity or vigorous-intensity activities accumulating at least 600 Metabolic Equivalent of Task-minutes (MET-minutes) per week [9].

### Energy expenditure

Time spent in each activity level, i.e. of vigorous activity, moderate activity, or walking is multiplied with the Metabolic Equivalent of Task (MET), which have previously been set to 8.0 for vigorous intensity, 4.0 for moderate intensity and 3.3 for walking [9, 10]. By multiplying the MET-value with number of days and minutes performed, weekly amount of each activity level can be calculated and expressed as MET-minutes. Further, by adding MET-minutes for each activity level, total MET-minutes per week can be assessed.

### Sedentary

Individuals were asked to recall the amount of time they had been sitting during a typical day for the last seven days.

### Spare time activity level

An additional item asked about individuals’ spare time activity level. Individuals categorized their spare time activity level during the last 12 months into one of four different activity levels. This question was dichotomized into low (“spending spare time mostly sedentary” and “light exercise at least 2 h per week”) and high activity (“moderate exercise once to twice per week, at least 30 min per session” and “moderate exercise at least three times per week, at least 30 min per session”).

### Sporting activities

The individuals were asked if they currently performed any exercise- or competitive sporting activities, which they answered as yes or no. Type of activity was not recorded.

### Anthropometrics

Data on body height and weight were self-reported.

### Radiology

Radiographic information was collected from the routine clinical examinations taken in conjunction to the survey. Cobb angles [11] median (25^th^, 75^th^ percentile) were for the subgroups as follows: Untreated 28 (19, 37) degrees, ongoing brace- treatment group 27 (23, 33) degrees, previously braced 36 (27, 44) degrees, and for the surgically treated 21 (17, 26) degrees. The number of fused vertebra was nine (seven, ten) for the surgically treated.

### Statistics

Descriptive data is presented as median (25^th^, 75^th^ percentile) or number (%). The distribution of continuous variables was checked by visual estimation. The variable MET minutes/week was skewed. A more normal distribution was achieved after log transformation with base 10, and the transformed variable was used in the statistical analyses. For group comparisons, analysis of covariance (ANCOVA) was used. Adjustments were made for age (as a continuous variable) or for sex and age and the corresponding *p*-value was presented. The groups of surgically-treated patients and brace-treated patients were dichotomized according to median time elapsed from surgery or cessation of brace treatment for comparison of physical activity level. A p-value of less than 0.05 was considered significant.

## Results

The group of adolescents with idiopathic scoliosis had a higher proportion of females and was older and taller than the individuals without idiopathic scoliosis (Table 1). When comparing adolescents with idiopathic scoliosis and without, there were no significant differences in the proportion achieving at least moderate activity level during a week (*p* = 0.15) or amount of MET-minutes/week (*p* = 0.13; Table 2). No differences were seen for time spent sitting (*p* = 0.81) or spare time activity level (*p* = 0.31; Table 2). The proportion participating in sporting activities did not differ between patients and controls (*p* = 0.28; Table 2).

**Table 1:** Descriptive statistics for individuals with and without idiopathic scoliosis. Data is reported as median (25th, 75th percentile) or number (%). Statistical analyses were performed with ANCOVA. *P* = level of significance

	Idiopathic scoliosis *n* = 239	No scoliosis *n* = 58	*P*
Females (%)	203 (85)	36 (61)	<0.001
Age (years)	16.0 (14.4, 17.6)	14.6 (12.8, 16.3)	<0.001
Weight (kg)	54 (50, 62)	52 (46, 65)	0.80^a^
Height (cm)	168 (162, 173)	165 (158, 174)	0.01^a^
Body Mass Index (kg/m^2^)	19.3 (17.9, 21.2)	19.4 (18.0, 22.2)	0.25^a^

^a^Adjusted for age and sex

**Table 2:** Physical activity in individuals with and without idiopathic scoliosis. Data is presented as median (25th, 75th percentile) or number (%). Statistical analyses were performed with ANCOVA, adjusted for age and sex. MET = Metabolic Equivalent of Task. *P* = level of significance

	Idiopathic scoliosis *n* = 239	No scoliosis *n* = 58	*P* (adjusted)
Achieving at least moderate activity level	180 (75 %)	49 (85 %)	0.14
Vigorous activity (days)^a^	1 (0, 3)	2 (0, 3)	0.20
Vigorous activity per day (minutes)^b^	60 (35, 90)	60 (35, 90)	0.22
Moderate activity (days)^a^	2 (0, 3)	2 (1, 4)	0.56
Moderate activity per day (minutes)^b^	43 (0, 90)	60 (11, 120)	0.12
Walking (days)^a^	5 (4, 7)	5 (4, 7)	0.21
Walking per day (minutes)^b^	40 (20, 60)	30 (20, 60)	0.76
MET-minutes/week	1977 (840, 3777)	2120 (887, 4598)	0.11
Time spent sitting (minutes/week)	420 (300, 540)	380 (300, 540)	0.94
Spare time high activity level	132 (55 %)	36 (62 %)	0.37
Exercise- or competitive sporting activities	125 (52 %)	36 (62 %)	0.28

^a^Time spent in activity for at least 10 min per day

^b^Time spent in activity less than 10 min was counted as zero. Activities exceeding 180 min per day were re-coded to 180 min

### Subgroup analysis

The group with ongoing brace-treatment was younger than the other groups (Table 3). The previously-braced group spent less time in vigorous activity than the other groups (*p* = 0.020; Table 4). The previously-braced and the surgically-treated group spent less time in moderate activity (*p* = 0.031) and had a lower spare time activity level (*p* = 0.046) compared to the other groups (Table 4). Previously-braced individuals reported lower MET-minutes/week when compared to the other groups (*p* = 0.031) (Table 4). The other physical activity variables did not differ between the groups (Table 4).

**Table 3:** Descriptive statistics for the different subgroups of individuals with scoliosis. Data is reported as median (25th, 75th percentile) or number (%). Statistical analyses were performed with ANCOVA

	Untreated *n* = 88	Ongoing brace *n* = 36	Previously braced *n* = 43	Surgically treated *n* = 72	*P*
Females (%)	70 (80)	33 (92)	39 (91)	61 (85)	0.22
Age (years)	15.7 (14.4, 17.3)	13.9 (13.0, 15.2)	17.1 (15.7, 18.3)	16.9 (14.9, 18.1)	<0.001
Weight (kg)	54 (50, 64)	51 (45, 55)	55 (51, 64)	55 (50, 63)	0.50
Height (cm)	169 (163, 173)	163 (158, 167)	170 (165, 175)	168 (162, 174)	0.18
Body Mass Index (kg/m^2^)	19.2 (18.0, 21.2)	18.6 (17.6, 20.3)	19.6 (18.1, 20.7)	19.5 (17.8, 21.8)	0.93^a^

^a^Adjusted for age

**Table 4:** Physical activity level for the individuals with idiopathic scoliosis, divided into untreated, brace treated (ongoing), previously brace treated, and surgically treated. Data is presented as median (25th, 75th percentile) or number (%). Statistical analyses were performed with ANCOVA, adjusted for age. MET = Metabolic Equivalent of Task. *P* = level of significance

	Untreated *n* = 88	Ongoing brace *n* = 36	Previously braced *n* = 43	Surgically treated *n* = 72	*P* (adjusted)
Achieving at least moderate activity level	69 (78 %)	30 (83 %)	28 (65 %)	53 (74 %)	0.11
Vigorous activity (days)^a^	2 (0, 3)	2 (0, 5)	1 (0, 3)	1 (0, 3)	0.050
Vigorous activity per day (minutes)^b^	60 (30, 75)	60 (45, 90)	45 (25, 60)	60 (58, 100)	0.020
Moderate activity (days)^a^	2 (0, 4)	2 (1, 4)	1 (0, 2)	2 (0, 3)	0.15
Moderate activity per day (minutes)^b^	60 (0, 113)	60 (15, 98)	25 (0, 60)	28 (0, 95)	0.031
Walking (days)^a^	6 (5, 7)	5 (4, 7)	5 (3, 7)	6 (4, 7)	0.49
Walking per day (minutes)^b^	40 (20, 79)	30 (20, 60)	30 (20, 60)	60 (20, 90)	0.85
MET-minutes/week	2160 (794, 3797)	2106 (990, 4480)	989 (661, 2706)	2055 (1010, 4026)	0.031
Time spent sitting (minutes/week)	420 (300, 540)	480 (285, 540)	480 (360, 600)	360 (210, 480)	0.14
Spare time high activity level	52 (59 %)	26 (72 %)	21 (49 %)	33 (46 %)	0.046
Exercise- or competitive sporting activities	46 (52 %)	26 (72 %)	20 (47 %)	33 (46 %)	0.20

^a^Time spent in activity for at least 10 min per day ^b^Time spent in activity less than 10 min was counted as zero. Activities exceeding 180 min per day were re-coded to 180 min

When dividing the surgically-treated individuals according to median time (1.5 years) after surgery, no significant differences were found for the different variables concerning physical activity (all *p* > 0.05). When dividing the previously brace-treated according to median time elapsed from treatment cessation (1.6 years), no significant differences were found in any of the variables except for spare time activity level. The individuals who recently terminated bracing more frequently reported themselves in the higher category of activity level in their spare time (*p* = 0.048).

There was no difference in the ongoing brace-treatment group regarding any variable when comparing those with nighttime brace and fulltime brace (all *p* > 0.05).

## Discussion

This study did not show any difference in self-reported physical activity level in adolescents with idiopathic scoliosis compared to individuals without idiopathic scoliosis.

To the best of our knowledge, this is the first study describing physical activity level in adolescents with idiopathic scoliosis that includes a comparison with a representative group of adolescents without idiopathic scoliosis. Our study documented all types of physical activity, including sporting activities. Besides describing the amount of physical activity in children with idiopathic scoliosis, we also speculate about a possible explanation for the previously reported findings of low bone density in children with scoliosis [5]. One limitation with the questionnaire used in the current study is that high impact activity was not specifically addressed, which is the type of activity that probably has the largest effect on bone density. Nevertheless, general physical activity has been shown to have a positive effect on bone density [12, 13].

We thought that brace wear may restrict physical activity, especially in daily life; this did not seem to be the case. Our findings are supported by other studies, reporting that adolescents treated with bracing do not get restricted in sporting activities [14] daily step activity [15], or oxygen consumption [2, 16]. Our group with ongoing brace-treatment had a higher participation rate in sports and a higher activity level in spare time, but there was no difference in MET-minutes/week compared to the other groups. Since MET-minutes are calculated solely based on the intensity items in IPAQ-SF, and not on the items regarding sports and spare time activities, it is therefore possible for discrepancy to exist.

There were no differences between the scoliosis subgroups regarding the primary outcome variable, but previously brace-treated individuals tended to have lower levels in some of the secondary outcome variables. The reason for this finding is not clear. Even if individuals in the previously-braced group were slightly older than the others, the difference to the surgically-treated group was only marginal, and these had an activity level comparable to the younger individuals in the ongoing brace-treatment group and the untreated individuals. In addition, all analyses were adjusted for age, used as a continuous variable in the analyses.

The previously-braced and the surgically-treated groups comprised the oldest individuals. The ongoing brace-treatment group and the untreated individuals had higher activity level. An age-effect can be considered since older adolescents might have less time for physical activity due to higher work-load in school, work etc. Another explanation might be that overestimation of physical activity in younger individuals is more frequent, which has been reported in previous studies [17, 18].

The long term effect of scoliosis on general health has been addressed by others [19, 20]. A cross-sectional study comparing sports activities among non-operatively and operatively treated individuals with scoliosis found that scoliosis patients were more restricted in their sporting activities compared to controls at middle-age [4].

Despite fusion of a significant number of vertebral levels, surgery had little impact on physical activity in this study. Time from surgery did not affect the results. It is also our impression that surgically-treated adolescents usually have recovered within a few months from surgery.

### Study limitations

This study has a number of limitations. The cross-sectional study design itself limits the conclusion to descriptive findings of physical activity levels in the different groups, but the study includes both untreated and treated individuals, as well as a population-based sample of adolescents without scoliosis. Participation rate was good for the patients (85 %), but substantially lower for the individuals without idiopathic scoliosis (12 %). Individuals without idiopathic scoliosis were selected from the population, which is a more difficult approach than to, for example, recruit individuals attending the hospital for other reasons than scoliosis. Still, we believed that this approach would give us a more representative cohort. Younger individuals without idiopathic scoliosis, aged 15 years or younger, were especially difficult to recruit. One reason could have been that adolescents less than 15 years of age required parents’ approval to be included in the study.

Despite the low participation rate, it seems that our non-scoliosis sample was fairly representative for the following reason. In a previous study, physical activity was assessed among 3051 invited adolescents across Europe [21] with a participation rate of 86 %. A total of 3546 adolescents were eligible from the beginning, which gave a high rate of participation (86 %). Their sample is considered to be representative due to inclusion of different countries and a high participation rate. The authors assessed physical activity with the long form of the IPAQ and made some modifications to optimize the questionnaire for adolescents. In their results, calculation of moderate physical activity level in mean minutes per week was 493, which is identical to the result in our non-scoliosis population. Moreover, representativeness, in a case-control study such as ours, may not be necessary other than that controls should not have the studied trait [22].

The IPAQ-SF has been compared to other questionnaires, accelerometers and general fitness. It has also been validated in different populations, and reports suggest it as a good tool for surveillance of physical activity. A weakness is that subjects answering the questionnaire easily overestimate physical activity [23–26]. However, even if physical activity may be overestimated with the IPAQ-SF, we do not believe there is a systematic bias between the groups. Both individuals with and without idiopathic scoliosis answered the same questionnaire which makes the comparisons feasible. Another possible weakness of the questionnaire is the fact that it does not take into account specific types of activities. Furthermore, there is a ceiling effect when re-coding values according to guidelines when managing data.

The questionnaire has been validated in populations 15 to 69 years of age [9]. It may be considered a limitation that some participants in the current study were younger than fifteen. Recent reports have discouraged the use of IPAQ-SF in adolescents under 15 years due to its overestimation of physical activity when compared with accelerometer data [18, 27]. The majority of the participants in the current study were older than fifteen. Others have found correlations between physical activity assessed with IPAQ-SF and heart rate and blood pressure in twelve to 17 year olds, indicating validity also in age groups similar to the present study [28].

Scoliosis has been suggested to be aggravated due to osteopenia [5]. Physical activity may prevent osteopenia [12, 13]. From a general health perspective, there is some room for improvement, since about one fifth of individuals with and without idiopathic scoliosis did not reach the recommendations of performing moderate and vigorous activities at least 60 min a day [29].

## Conclusion

In this study, our results indicate no difference in self-reported physical activity level among adolescents with idiopathic scoliosis and a representative sample of adolescents without scoliosis. We found that individuals with idiopathic scoliosis who were being treated with bracing, or had been surgically treated, were not restricted in their physical activity.
